# Supplementary material for: Content-rich biological network constructed by mining PubMed abstracts
Source: BMC Bioinformatics. 2004 Oct 8;5:147. doi: 10.1186/1471-2105-5-147 (PMC528731; doi:10.1186/1471-2105-5-147)
Supplement: Additional File 2 — The original results of the above study (non-essential files are deleted to keep the file size under the limit set by BMC bioinformatics). [file 1471-2105-5-147-S2.bz2 › chilibotAdditionalFile2/dip30/241ID8816500E790/html/BIK_BCL2.html]

 


 **BIK** and **BCL2** 
  
Found 354 abstracts in PubMed, retrieved 30.  
 

 What does Google say? 
 PDF only 
| .edu only 

---

**Interactive relationship** (e.g. stimulation, inhibition, etc)

**Neutral relationship**- Bax  **Bcl2**  associated X protein is an apoptosis inducing  [ **BIK** ]  protein that participates in cell death during normal development and in various diseases.  Ref: 12732850 Nature, 2003
- Immunoprecipitation studies revealed that Nbk  [ **BIK** ]  interacts with Bcl x L and Bcl 2  [ **BCL2** ]  but not with Bax.  Ref: 12853473 EMBO J< MedlineTA>EMBO J, 2003
- The bcl 2  [ **BCL2** ]  homologue antagonist killer BAK is a potently apoptosis inducing  [ **BIK** ]  gene and plays an important role in modulating apoptosis in epithelial cells.  Ref: 12767516 Cancer Lett, 2003
- **BIK** , a BH  **Bcl2**  homology domain 3 only protein, is a proapoptotic member of the  **BCL2**  family.  Ref: 12874789 Genes Chromosomes Cancer, 2003
- A 24 h exposure of PC 3 and LNCaP cells to an apoptosis inducing  [ **BIK** ]  concentration of AITC 20 micro M resulted in a significant decrease 31 68% in the levels of anti apoptotic protein Bcl 2  [ **BCL2** ]  in both cell lines, and approximately 58% reduction in Bcl X L protein expression in LNCaP cells.  Ref: 12771033 Carcinogenesis, 2003

**Non-interactive relationship** (e.g. studied together, co-existance, homology, etc.)

- To examine p75 NTR dependent apoptosis in tumor cells, we demonstrated that a dose dependent increase in p75 NTR expression was associated with a concomitant increase in the mitochondrial proapoptotic effector proteins Bad, Bax and  **Bik**  and a decrease in the mitochondrial prosurvival effector proteins phospho Bad, Bcl 2  [ **BCL2** ]  and Bcl x L.  Ref: 12672029 Int J Cancer, 2003
- Nbk  [ **BIK** ]   **Bik**  natural born killer Bcl 2  [ **BCL2** ]  interacting killer  [ **BIK** ]  is a tissue specific BH3 only protein whose molecular function is still largely unknown.  Ref: 12853473 EMBO J< MedlineTA>EMBO J, 2003
- To define molecular requirements for ErPC induced apoptosis, activation of caspases and 3 and cleavage of the caspase 3 substrates PARP and ICAD were tested in normal Jurkat T cells, Jurkat cells resistant to death receptor CD95 or TNFalpha related apoptosis inducing  [ **BIK** ]  ligand TRAIL induced apoptosis, Jurkat cells lacking caspase 8 or Fas associated death domain FADD Jurkat cells expressing a dominant negative caspase 9 or overexpressing Bcl 2  [ **BCL2** ]  as well as BJAB B lymphoma cells expressing a dominant negative FADD FADD DN.  Ref: 12730676 Oncogene, 2003
- Induction of cell death by the BH3 only Bcl 2  [ **BCL2** ]  homolog Nbk  [ **BIK** ]   **Bik**  is mediated by an entirely Bax dependent mitochondrial pathway.  Ref: 12853473 EMBO J< MedlineTA>EMBO J, 2003
